# The LncRNA Expression Profile and Regulatory Network of Microsporidian During the Infection of Western Honeybee

**DOI:** 10.3390/ani16132102

**Published:** 2026-07-07

**Authors:** Wei Wang, Jiarun Yang, Kaiyao Zhang, Shujun Yuan, Mengyuan Dai, Yuchen Sun, Dafu Chen, Rui Guo, Jianfeng Qiu

**Affiliations:** 1 College of Bee Science, Fujian Agriculture and Forestry University, Fuzhou 350002, China; 18151089132@163.com (W.W.); 13064033836@163.com (J.Y.); kaiyao1223@126.com (K.Z.); yuanshujun1123@163.com (S.Y.); 18050348332@163.com (M.D.); sunyuchen0917@163.com (Y.S.); dfchen826@fafu.edu.cn (D.C.); 2 National & Local United Engineering Laboratory of Natural Biotoxin, Fuzhou 350002, China; 3 Apitherapy Research Institute, Fujian Agriculture and Forestry University, Fuzhou 350002, China

**Keywords:** *Vairimorpha ceranae*, long non-coding RNA, *Apis mellifera*, competing endogenous RNA, host–pathogen interaction, nosemosis

## Abstract

*Vairimorpha ceranae* is a fungal pathogen that infects the honeybee midgut and poses a serious threat to colony health. However, the role of long noncoding RNAs (lncRNAs) of *V*. *ceranae* in its infection of the host remains poorly understood. Using lncRNA-seq data from the midguts of *Apis mellifera* workers at 7 and 10 days post-inoculation with *V*. *ceranae* (NcT1L and NcT2L groups), along with controls inoculated with spores (NcCKL group), we performed transcriptome-wide identification and structural characterization of lncRNAs. We identified lncRNAs in *V*. *ceranae* and analyzed the regulatory network of the differentially expressed lncRNAs (DElncRNAs). A total of 27 *V*. *ceranae* lncRNAs were identified in the midguts. The 19, 21, and 4 DElncRNAs were identified in the NcCKL vs. NcT1L, NcCKL vs. NcT2L, and NcT1L vs. NcT2L comparison groups. These DElncRNAs were predicted to regulate 26, 27, and 2 upstream/downstream genes. Furthermore, 15, 23, and 4 DElncRNAs were found to target 195, 211, and 94 miRNAs, which in turn targeted 204, 216, and 73 mRNAs into the respective comparisons. The ceRNA network prediction revealed that DElncRNAs, miRNAs and mRNAs form a complex regulatory network. This study presents the expression profile of lncRNAs during *V*. *ceranae* infection and highlights their potential regulatory functions in pathogenesis. Our findings provide new molecular insights into host–pathogen interactions at the RNA level and establish a foundation for developing targeted strategies to control nosemosis.

## Simple Summary

The microsporidian parasite *Vairimorpha ceranae* infects the midgut of honeybees and causes a disease called nosemosis, which threatens bee colonies and, consequently, global food production. In this study, we investigated tiny regulatory molecules, called long non-coding RNAs, that the parasite produces during infection. Using advanced sequencing techniques, we identified 27 such molecules in the parasite. Their activity changes as the infection progresses: some become more active early during invasion, while others peak later when the parasite multiplies. We also built detailed maps showing how these molecules interact with other genetic components to control the infection process. Our findings provide the first comprehensive view of how *V*. *ceranae* uses long non-coding RNAs to regulate its own development and interaction with the host. This knowledge opens new avenues for designing targeted strategies to control nosemosis and protect honeybee health.

## 1. Introduction

*Apis mellifera* is a crucial pollinator in natural ecosystems and agricultural landscapes, playing a vital role in maintaining flowering plant diversity and ensuring global food security [1]. *Vairimorpha ceranae*, an obligate intracellular microsporidian parasite that primarily infects honey bees, causes nosemosis, posing a serious threat to colony health and the sustainable development of apiculture [2]. *Vairimorpha ceranae* was formally identified and described by Ingemar Fries in 1996 from *Apis cerana* [3]. *Vairimorpha ceranae* has largely supplanted *Vairimorpha apis* to become the predominant causative agent of honey bee nosemosis worldwide [4,5]. This pathogen is characterized by high latency, rapid transmission, and specific tropism for the midgut epithelial cells of honey bees, leading to severe impairments in nutrient absorption, immune suppression, and gut microbiota dysbiosis, ultimately contributing to colony collapse [6]. Temperature is regarded as an important environmental factor influencing the successful colonization of *V. ceranae* in the bee gut. Low temperatures have a significant impact on the spores of microsporidia [7]. However, in South-Western Siberia, where the average January temperature is below −18 °C, both *V. ceranae* and *V. apis* persist [6].

Long non-coding RNAs (lncRNAs), defined as non-coding RNA transcripts exceeding 200 nucleotides in length, do not encode proteins but play pivotal roles in regulating gene expression [8,9,10]. They influence host cell physiology and pathogen virulence through diverse mechanisms, including epigenetic regulation, cell cycle control, and immune responses [11]. Advances in high-throughput sequencing technologies, bioinformatics tools, and analytical methods have led to the identification of an increasing number of lncRNAs. Experimental studies have demonstrated their regulatory importance in various biological processes such as epigenetics, dosage compensation, genomic imprinting, embryonic development, cell cycle progression, and cellular differentiation [10].

LncRNAs have been identified in fungi such as *Saccharomyces cerevisiae* [12,13], *Paracoccidioides brasiliensis* [14], and *Trichoderma reesei* [15]. Recent studies have also revealed regulatory roles of lncRNAs in bacterial, fungal, and viral pathogens. In human-pathogenic fungi, the lncRNA RZE1 controls morphological transition in *Cryptococcus neoformans* [16], and a genome-wide screen in *Aspergillus fumigatus* identified 60 condition-dependent lncRNA mutants modulating antifungal drug sensitivity [17]. In the intracellular bacterium *Coxiella burnetii*, the host lncRNA CYP1B1-AS1 promotes pathogenesis by inhibiting ROS and host cell death [18]. In *Mycobacterium tuberculosis*, the lncRNA Lnc-Gm5532 induces cuproptosis to enhance intracellular survival [19]. In plant pathogens, *Fusarium graminearum* lncRsp1 regulates ascospore discharge and virulence [20], while FG-lncRNA06490 mediates mycotoxin production and full pathogenesis [21]. In viruses, Epstein–Barr virus encodes its own lncRNAs that control the viral lytic switch [22], and the host lncRNA LINC2781 enhances antiviral immunity against coxsackievirus B5 infection by activating the JAK-STAT pathway [23]. A functional lncRNA 6140 was discovered in the pathogen of bee chalkbrood disease, *Ascosphaera apis*. It promotes infection through the milr5658-x-ATPase axis [24]. However, lncRNA research within the phylum Microsporidia remains extremely limited [25,26]. Furthermore, emerging evidence indicates that lncRNAs can regulate fungal hyphal growth, sporulation, and virulence [16,17,27]. Guo et al. [25] first identified lncRNAs in the spores of *V. ceranae*. Zhou et al. [28] constructed a ceRNA network for *V. ceranae*. Guo et al. [29] constructed the full-length transcriptome of *V. ceranae*, and identified numerous virulence factor-associated genes and transcripts, as well as lncRNAs. However, the functional roles of lncRNAs in the pathogenesis of *V*. *ceranae* infection are still largely unexplored.

Microsporidia are characterized by their extremely compact genomes, which encode roughly 2280 proteins [30]. Considering that lncRNAs are capable of modulating multiple downstream targets with modest metabolic overhead, they may serve as critical regulatory elements in microsporidia, contributing to the expansion of regulatory complexity, the conservation of cellular energy, and the orchestration of host infection processes. This study employs deep sequencing and omics approaches to analyze the differential expression profiles of lncRNAs in *V*. *ceranae* during its infection of *A. mellifera*. We systematically investigate the potential functions of differentially expressed lncRNAs (DElncRNAs) through analyses of cis- and trans-regulation, antisense lncRNA interactions, and competing endogenous RNA (ceRNA) networks. The aim is to elucidate the molecular mechanisms underlying *V*. *ceranae* infection in *A. mellifera* workers from a lncRNA omics perspective.

## 2. Materials and Methods

### 2.1. Biological Materials

The *A. mellifera* workers were obtained from the College of Bee Science and Biomedicine, Fujian Agriculture and Forestry University, Fuzhou city, China. *Vairimorpha ceranae* infected worker bees were collected from a diseased colony at the Jingxi Yuan’an Apiary in Minhou County, Fuzhou City, China.

### 2.2. Purification of V. ceranae Spores

Fresh spores were isolated from naturally infected bees collected in Fuzhou, Fujian Province, China, following a modified protocol based on Cornman et al. [31] and previous work [27,32]. Briefly, bees were anesthetized at 4 °C for 5 min, after which midguts were dissected and homogenized in distilled water. The homogenate was filtered through four layers of sterile gauze and centrifuged three times at 6000× *g* for 5 min each. Spores were collected from the sediment, resuspended, and purified by centrifugation at 18,000× *g* for 90 min at 4 °C on a discontinuous Percoll gradient (25–100%). The spore pellet was carefully collected with a sterile syringe and subjected to a second Percoll gradient centrifugation to obtain purified spores, which were flash-frozen in liquid nitrogen and stored at 4 °C.

### 2.3. Spore Inoculation and Sample Preparation

Newly emerged honeybees were obtained by maintaining sealed brood frames in an incubator at 34 ± 0.5 °C and 50% RH. Upon emergence, workers were collected and housed in cages (20 bees per cage) at 32 ± 0.5 °C and 50% RH, with ad libitum access to 50% (w/w) sucrose solution. After 24 h, bees were starved for 2 h, and individually inoculated with 5 μL of 50% sucrose solution containing 1 × 10^6^ spores of *V. ceranae*. Midguts were sampled at 7 and 10 days post-inoculation (dpi), flash-frozen in liquid nitrogen, and stored at −80 °C for subsequent sequencing and molecular analyses. The *V. ceranae*-inoculated groups at 7 and 10 dpi were labeled NcT1L and NcT2L, respectively. Each group had three biological replicates, and each biological replicate consisted of six bees. The control group consisted of pure *V. ceranae* spores (NcCKL) and was divided into three replicates.

### 2.4. Total RNA Extraction and LncRNA Sequencing

Total RNA was extracted from the aforementioned midgut samples using TRIzol reagent (Invitrogen, Carlsbad, CA, USA). Strand-specific cDNA libraries were then constructed. The prepared libraries were sequenced on an Illumina HiSeq™ 4000 platform (Denovo, Guangzhou, China), yielding mixed transcriptome data from both the host and the pathogen, which included mRNA, miRNA, and lncRNA sequences. The corresponding raw sequencing data have been deposited in the NCBI database (https://www.ncbi.nlm.nih.gov/sra (accessed on 5 May 2026)) and linked to BioProject accession number: PRJNA406998. Previously, we investigated the host transcriptional response to *V. ceranae* infection [27,32]. LncRNAs derived from *V. ceranae* spores, as well as those originating from the spores during host infection, have not been reported.

### 2.5. Quality Control of LncRNA-seq Data and Screening of V. ceranae LncRNA Data

Raw reads obtained from sequencing were subjected to sequencing saturation analysis. Adaptor sequences, low-quality reads, and reads containing ambiguous nucleotides were removed using Perl scripts to obtain clean reads. These clean reads were then aligned to a ribosomal RNA database using Bowtie [33] with zero mismatches allowed. The remaining reads that did not map to the ribosomal database were subsequently aligned to the *A. mellifera* reference genome (assembly AmeI_4.5) using TopHat2 [34]. Reads successfully mapped to the host genome were filtered out. The unmapped reads were further aligned to the *V. ceranae* reference genome (assembly ASM98816v1). Reads uniquely mapping to the *V. ceranae* genome were retained as the pathogen-specific dataset for subsequent analysis.

The coding potential of the assembled transcripts was predicted using both the CPC (Coding Potential Calculator) [35] and the CNCI (Coding-Non-Coding Index) [36]. Transcripts consistently classified as non-coding by both tools were defined as high-confidence lncRNAs. The lncRNA transcriptome datasets derived from *V. ceranae* spores isolated from worker bee midguts at 7 dpi and 10 dpi post-inoculation were designated as NcT1L and NcT2L, respectively.

### 2.6. Structural Characterization of LncRNAs

Based on the lncRNA omics data corresponding to NcCKL, NcT1L, and NcT2L, lncRNAs within each dataset were identified using CPC [35] and CNCI [36]. The exon length, exon number, and transcript length of lncRNAs and mRNAs were separately calculated and statistically summarized for the NcCKL, NcT1L and NcT2L datasets.

### 2.7. Antisense lncRNA Analysis

The RNAplex software (version 0.2) [37] was employed to predict complementary binding interactions between antisense lncRNAs and mRNAs. The minimum free energy was calculated based on the thermodynamic stability of the predicted structures to identify the optimal base-pairing relationships.

### 2.8. Differential Expression Analysis of LncRNAs

The expression level of each lncRNA was normalized to FPKM (Fragments Per Kilobase of exon model per Million mapped fragments) values. Differential expression analysis of lncRNAs was performed using the OmicShare platform (https://www.omicshare.com/ (accessed on 5 May 2026)) with its default parameters. Specifically, the platform implements the edgeR package (v3.28.1) for count-based differential expression analysis, with normalization using the TMM (trimmed mean of M-values) method. A lncRNA was considered differentially expressed if it met both of the following criteria: (i) |log_2_ (fold change)| ≥ 1, and (ii) false discovery rate (FDR)-adjusted *p* value < 0.05, where the Benjamini–Hochberg method was applied for multiple testing correction. Venn analysis of DElncRNAs was conducted using the relevant tools of the Biomic Cloud Platform (http://www.biocloud.net/gongju (accessed on 5 May 2026)).

### 2.9. Cis/Trans-Acting Analysis of DElncRNAs

The differential expression of LncRNA affected the changes in upstream and downstream gene expression, and the protein-coding genes within 10 kb were screened according to the gene position of lncRNA [38,39].

Target genes of the DElncRNAs were predicted based on the correlation analysis of expression levels between lncRNAs and protein-coding genes across samples. A regulatory network was constructed according to the predicted lncRNA-mRNA targeting relationships and visualized using Cytoscape software (version 2.8) [40].

### 2.10. Analysis of the Competing Endogenous RNA (ceRNA) Regulatory Network

The previously performed sRNA-seq (small RNA sequencing) on purified *V. ceranae* spores, as well as on midguts of *A. mellifera* workers infected with *V. ceranae* at 7 dpi and 10 dpi post-inoculation, generating high-quality sRNA-seq data. The related raw data have been submitted to the NCBI database (https://www.ncbi.nlm.nih.gov/sra (accessed on 5 May 2026)) and linked to BioProject accession numbers: PRJNA395264 and PRJNA395137 [27,32]. These high-quality miRNA omics data provide a reliable foundation for the target prediction of DElncRNAs (differentially expressed lncRNAs) and the construction and analysis of regulatory networks in this study.

The miRNA binding sites on DElncRNAs and the target mRNAs of these miRNAs were predicted using RNAhybrid (version 2.1.2) [41], miRanda (version 3.3a) [42], and TargetScan (version 7.0) [43] software. In order to minimize the incidence of false-positive predictions, parallel target predictions were conducted using three independent computational algorithms (RNAhybrid, Miranda, and TargetScan). Only those targets that were consistently identified by all three tools were retained as high-confidence candidate targets for downstream analyses. Based on these interactions, lncRNA-miRNA and lncRNA-miRNA-mRNA regulatory networks were constructed. The resulting ceRNA networks were visualized using Cytoscape [40].

### 2.11. RT-PCR Validation of Novel LncRNAs

Six novel lncRNAs (TCONS_00003693, TCONS_00003047, TCONS_00000755, TCONS_00003691, TCONS_00003749, and TCONS_00003694) were randomly selected from the prediction results for experimental validation by RT-PCR. Specific primers were designed using Primer Premier (version 5.0) software [44].

Total RNA was extracted from purified *V. ceranae* spores and from midguts of *A. mellifera* workers collected at 7 dpi and 10 dpi using an RNA extraction kit (TaKaRa, Dalian, China). First-strand cDNA was synthesized from the RNA templates using the HiScript 1st Strand cDNA Synthesis Kit (Vazyme, Nanjing, China). PCR amplification was performed on a T100 Thermal Cycler (Bio-Rad Laboratories, Hercules, CA, USA). The 10 μL reaction mixture contained: 1 μL template cDNA, 1 μL PCR mix, 1 μL each of forward and reverse primer (4 μmol/L), and 7 μL sterile water. The PCR protocol was as follows: initial denaturation at 95 °C for 5 min; 34 cycles of denaturation at 95 °C for 50 s, annealing at 55 °C for 30 s, and extension at 72 °C for 50 s; followed by a final extension at 72 °C for 5 min. The PCR products were separated by electrophoresis on a 1.5% agarose gel and visualized under UV light using a gel documentation system.

### 2.12. Validation of DElncRNAs by RT-qPCR

Five DElncRNAs (TCONS_00002929, TCONS_00003749, TCONS_00003691, TCONS_00002930, and TCONS_00002069) were randomly selected from the NcCKL vs. NcT1L comparison group, and an additional five DElncRNAs (TCONS_00002929, TCONS_00003749, TCONS_00003691, TCONS_00002930, and TCONS_00003047) were selected from the NcCKL vs. NcT2L comparison group for validation by RT-qPCR. Specific primers were designed using Primer Premier 5 (Table 1). Total RNA was extracted from purified *V. ceranae* spores and from midguts of infected bees at 7 dpi and 10 dpi using an RNA extraction kit (TaKaRa, Dalian, China). RNA was reverse-transcribed into first-strand cDNA using a cDNA synthesis kit (Vazyme, Nanjing, China) to serve as the template for RT-qPCR. RT-qPCR was performed on a QuantStudio 3 Real-Time PCR System (Thermo, Waltham, MA, USA) using the SYBR Green Dye kit (Vazyme, Nanjing, China) according to the manufacturer’s instructions. Three biological replicates were performed, with each replicate consisting of a pool of midguts from six bees. The 10 μL reaction mixture contained: 1 μL template cDNA, 1 μL each of forward and reverse primer (4 μmol/L), 1 μL SYBR Green Dye, and 7 μL DEPC-treated water. The thermal cycling conditions were: initial denaturation at 95 °C for 1 min; followed by 40 cycles of denaturation at 95 °C for 15 s and annealing/extension at 60 °C for 30 s. Each reaction was set up in three technical replicates. The relative expression levels of the target genes were calculated using the 2^−ΔΔCt^ method.

## 3. Results

### 3.1. Identification of LncRNAs in V. ceranae

**Figure 1:**
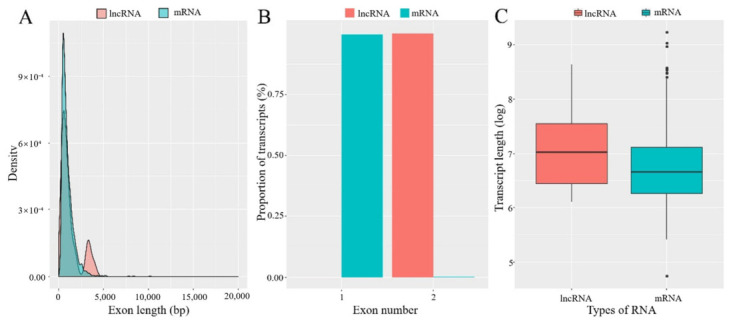
The lncRNA and mRNA characteristics of *V. ceranae*. (**A**) Exon length of lncRNAs and mRNA of *V. ceranae*. (**B**) Exon number of lncRNAs and mRNA of *V. ceranae*. (**C**) Transcript length of lncRNAs and mRNA of *V. ceranae*.

A total of 27 lncRNAs in *V. ceranae* were predicted. Comparative analysis revealed that the exon lengths of both lncRNAs and mRNAs were predominantly below 2500 bp, with only a minority of lncRNAs exhibiting exon lengths between 2500 and 5000 bp (Figure 1A). The vast majority of lncRNAs contained 2 exons, whereas most mRNAs contained only 1 exon (Figure 1B). Furthermore, the average transcript length of lncRNAs was longer than that of mRNAs (Figure 1C).

**Figure 2:**
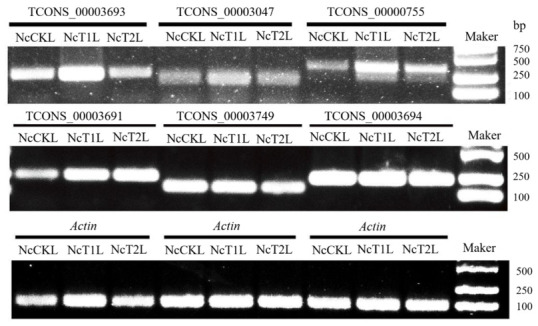
RT-PCR validation of lncRNAs in *V. ceranae*.

Six lncRNAs were randomly selected for RT-PCR verification. The results of agarose gel electrophoresis confirmed that specific bands were amplified for all the lncRNAs (Figure 2), which proved the actual expression of the lncRNAs predicted in this study.

**Figure 3:**
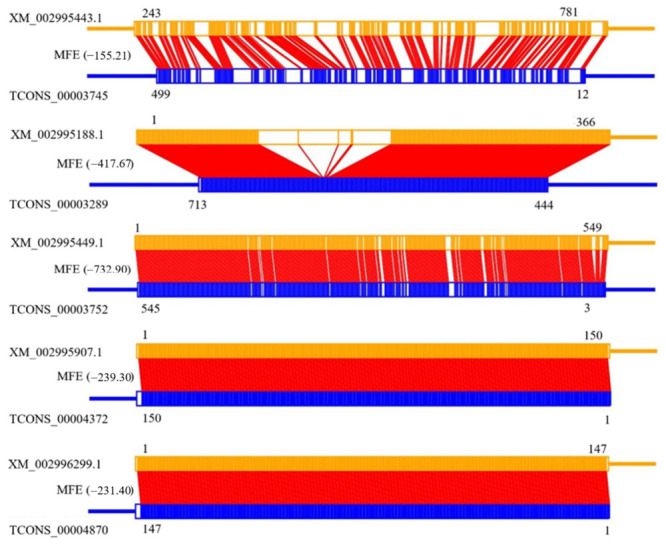
Prediction of pairings between antisense lncRNAs and corresponding sense strand mRNAs.

The five lncRNAs were identified as capable of binding complementarily to their corresponding sense mRNAs (Figure 3). These antisense lncRNAs were TCONS_00003745, TCONS_00003752, TCONS_00003289, TCONS_00004372, and TCONS_00004870, which putatively bind to the mRNAs encoding a Skn7 homolog (XM_002995443.1), hypothetical protein (XM_002995449.1), another hypothetical protein (XM_002995188.1), kinesin family protein (XM_002995907.1), and a Ricin B lectin homolog (XM_002996299.1), respectively.

### 3.2. LncRNA Expression Profiling and Differential Expression Analysis

**Figure 4:**
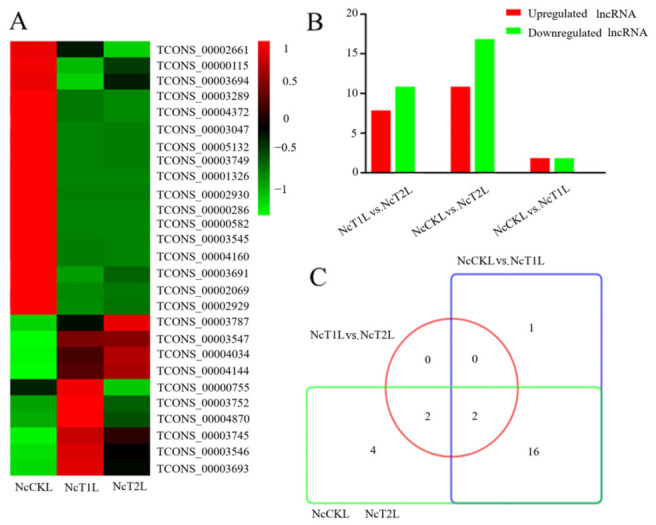
LncRNA expression level clustering analysis and DElncRNA analysis of *V. ceranae*. (**A**) LncRNA expression level clustering analysis. (**B**) DElncRNAs analysis. 4 ≥ |log_2_ (fold change)| ≥ 1, *p* values (Benjamini–Hochberg) < 0.05. (**C**) Venn analysis of DElncRNAs.

Expression clustering analysis indicated distinct expression patterns among *V. ceranae* lncRNAs during infection. Some lncRNAs exhibited higher expression levels in purified spores, which decreased during the infection of *A. mellifera* worker bees. Conversely, the expression levels of some other lncRNAs were elevated during infection compared to the spore stage, showing an initial increase followed by a decrease. Additionally, some lncRNAs exhibited low expression levels in spores, with their expression increasing at 7 dpi and then decreasing again at 10 dpi (Figure 4A).

Differential expression analysis identified 8 upregulated and 11 downregulated lncRNAs in the NcCKL vs. NcT1L comparison group. The NcCKL vs. NcT2L comparison group contained 7 upregulated and 16 downregulated lncRNAs. The NcT1L vs. NcT2L comparison group contained 2 upregulated and 2 downregulated lncRNAs (Figure 4B). Venn analysis of DElncRNAs across the three comparison groups revealed that 2 DElncRNAs were common to all three groups. Furthermore, 1 and 4 DElncRNAs were unique to the NcCKL vs. NcT1L and NcCKL vs. NcT2L comparison groups, respectively (Figure 4C).

**Figure 5:**
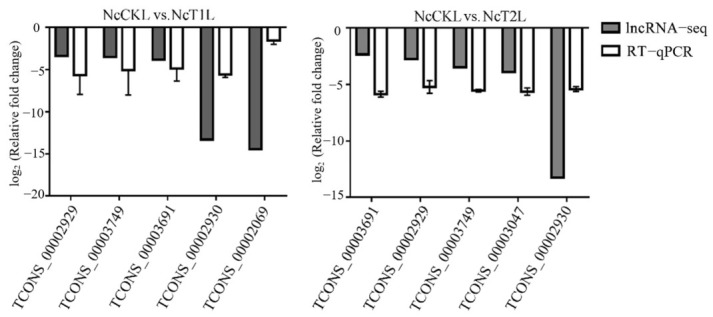
RT−qPCR confirmation of DElncRNAs.

Six DElncRNAs were randomly selected for validation using RT-qPCR. The results demonstrated that their expression trends were consistent with the transcriptome sequencing data (Figure 5), confirming the reliability and accuracy of the lncRNA omics data generated in this study.

### 3.3. The Cis/Trans-Acting of DElncRNAs

Cis-acting analysis predicted 26 potential target genes located within 10 kb upstream or downstream of 12 DElncRNAs in the NcCKL vs. NcT1L comparison, 27 target genes for 12 DElncRNAs in the NcCKL vs. NcT2L comparison, and two target genes for two DElncRNAs in the NcT1L vs. NcT2L comparison.

**Figure 6:**
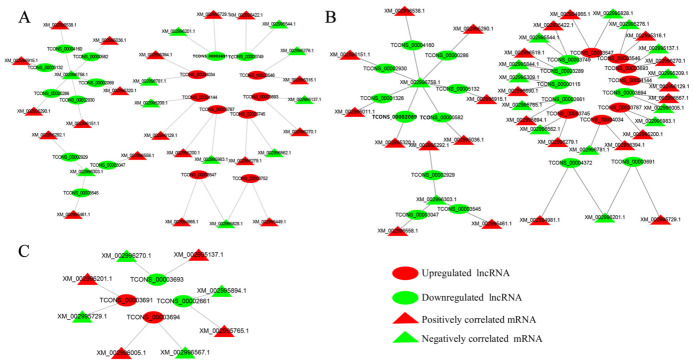
The predictive networks between *trans*-acting DElncRNAs and corresponding mRNAs in every comparison groups. (**A**–**C**) *Trans*-acting DElncRNAs and corresponding mRNAs in the NcCKL vs. NcT1L, NcCKL vs. NcT2L, and NcT1L vs. NcT2L comparison groups.

Trans-acting analysis further predicted that multiple DElncRNAs may co-regulate a single mRNA. The TCONS_00000286, TCONS_00002930, TCONS_00004160, TCONS_00002069, TCONS_00005132 and TCONS_00000582 were predicted to jointly regulate XM_002996758.1. More frequently, a single lncRNA shower correlated expression with one positively correlated mRNA and one negatively correlated mRNA. For example, TCONS_00003691 was predicted to regulate both XM_002995729.1 (positive correlation) and XM_002996201.1 (negative correlation) (Figure 6).

### 3.4. Prediction of the Regulatory Networks of DElncRNA

**Figure 7:**
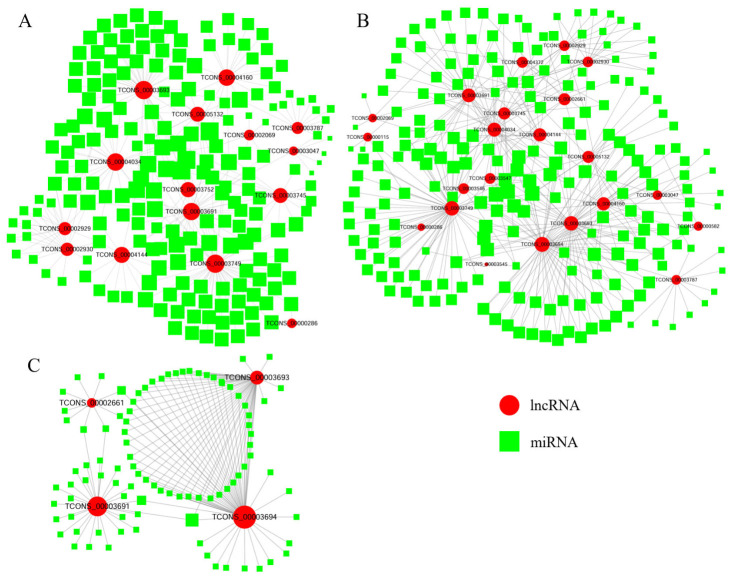
The predicted regulation networks between DElncRNAs and target miRNAs. (**A**–**C**) DElncRNAs and corresponding target miRNAs in the NcCKL vs. NcT1L, NcCKL vs. NcT2L, and NcT1L vs. NcT2L comparison groups.

**Figure 8:**
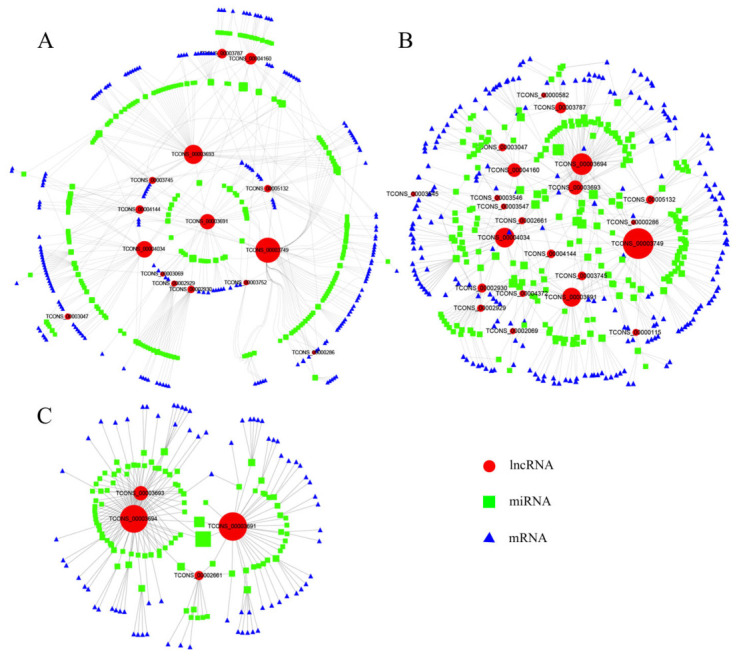
The predicted ceRNA regulatory networks of DElncRNAs. (**A**–**C**) DElncRNAs in the NcCKL vs. NcT1L, NcCKL vs. NcT2L, and NcT1L vs. NcT2L comparison groups.

In the NcCKL vs. NcT1L comparison group, 15 DElncRNAs were predicted to target 195 miRNAs (Figure 7A), which in turn were predicted to target 204 mRNAs (Figure 8A). In the NcCKL vs. NcT2L comparison, 23 DElncRNAs targeted 211 miRNAs (Figure 7B), which subsequently targeted 216 mRNAs (Figure 8B). For the NcT1L vs. NcT2L comparison, 4 DElncRNAs targeted 94 miRNAs (Figure 7C), and these miRNAs were predicted to bind 73 mRNAs (Figure 8C). Among the DElncRNAs common to all three comparison groups, TCONS_00003694 and TCONS_00003693 targeted the highest number of miRNAs (56 and 47, respectively). These results indicate that most lncRNAs may bind multiple miRNAs, and each miRNA may, in turn, regulate multiple mRNAs, forming complex regulatory networks.

**Figure A1:**
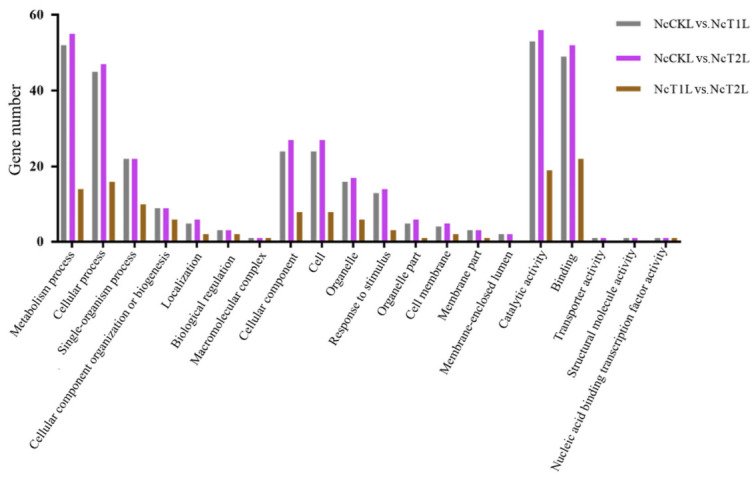
GO database annotation of target mRNAs in ceRNA regulatory networks.

The GO annotation of the mRNA targets of the miRNAs linked to the DElncRNAs was performed. For the NcCKL vs. NcT1L comparison, the top six GO functional terms with the highest number of annotated target mRNAs were catalytic activity (53), metabolic process (52), binding (49), cellular process (45), cell (24), and cellular component (24). Similarly, for the NcCKL vs. NcT2L comparison, the most enriched terms were catalytic activity (56), metabolic process (55), binding (52), cellular process (47), cell (27), and cellular component (27). In the NcT1L vs. NcT2L comparison, the top terms were binding (22), catalytic activity (19), cellular process (16), metabolic process (14), single-organism process (10), and cell (8) (Figure A1).

**Figure A2:**
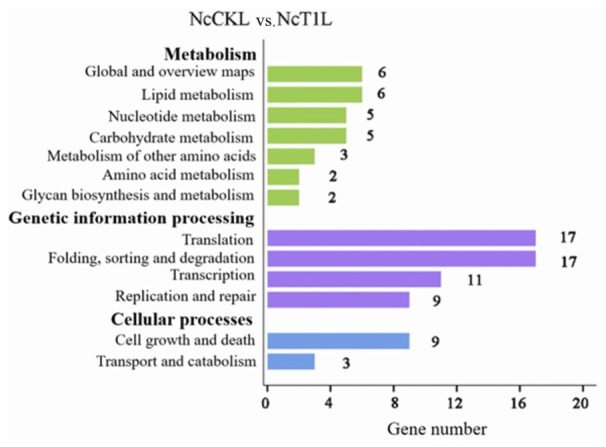

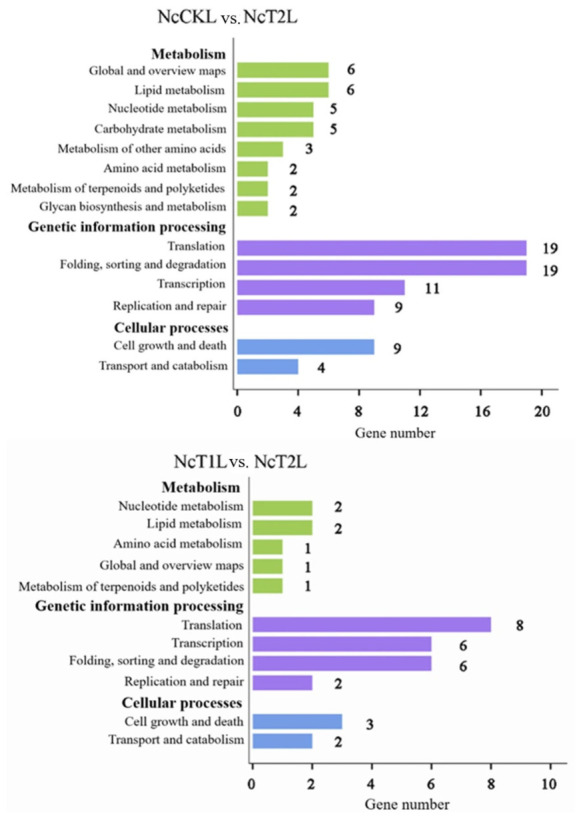
KEGG database annotation of target mRNAs in ceRNA regulatory networks.

KEGG pathway annotation analysis showed that for the NcCKL vs. NcT1L comparison, 48 target mRNAs were annotated to 25 metabolism-related pathways, 67 target mRNAs to 19 pathways associated with genetic information processing, and 17 target mRNAs to 4 pathways involved in cellular processes. For the NcCKL vs. NcT2L comparison, 50 target mRNAs were annotated to 26 metabolic pathways, 71 target mRNAs to 19 genetic information processing pathways, and 18 target mRNAs to 4 cellular process pathways. In the NcT1L vs. NcT2L comparison, 12 target mRNAs were mapped to 10 metabolic pathways, 23 target mRNAs to 13 genetic information processing pathways, and 6 target mRNAs to 4 cellular process pathways (Figure A2).

## 4. Discussion

LncRNAs regulate pathogenicity and host–pathogen interactions across diverse pathogens [45]. Yet for microsporidia—obligate intracellular parasites that devastate honeybee colonies—their lncRNA landscapes remained unexplored. The primary objective of this study was to screen and identify DElncRNAs of *V. ceranae* during its infection of honeybees, with the aim of preliminarily obtaining candidate lncRNAs that may play functional roles in the host infection process. Here we profiled lncRNAs of *V. ceranae* directly from infected *A. mellifera* midguts, identifying 27 high-confidence transcripts. Beyond cataloging, we uncovered multi-layered regulatory networks (antisense, cis, trans, ceRNA) that operate in a stage-specific manner during infection. In microsporidian-infected hosts, although hundreds to thousands of lncRNAs are detectable, most originate from the host, and only a limited number—ranging from dozens to several hundred—of predicted but uncharacterized microsporidian lncRNAs remain after host-genome filtering [29,46,47]. In our previous work, we employed PacBio single-molecule real-time (SMRT) sequencing to generate a full-length transcriptome of *V. ceranae*, which obtained merely 29 lncRNAs [29]. By comparison, 238 lncRNAs have been identified in Saccharomyces cerevisiae [48]. It is speculated that the low number of lncRNAs in *V. ceranae* may be attributed to its streamlined genome [31].

The *V. ceranae* lncRNAs possess more exons and longer transcripts than protein-coding mRNAs, a pattern opposite to that in model fungi [12,13]. Given the highly streamlined genome of microsporidia [49], this structural complexity may compensate for a reduced repertoire of protein regulators. In the present study, a limited number of lncRNAs were found to contain two exons. Unlike protein-coding genes, lncRNAs often rely on splicing for proper maturation, nuclear export, stability, and functional activity [50,51]. The core components of the spliceosome—including U2, U4, and U5 snRNAs as well as several key splicing factors—have been retained in microsporidian genomes [52]. Thus, while protein-coding genes have largely abandoned introns to reduce energy expenditure, lncRNAs may retain a structure similar to introns because their regulatory functions depend on splicing events. We speculated that longer transcripts and additional exons may enable interactions with multiple regulatory factors—an economical adaptation for tight genetic control within a compact genome.

The *V. ceranae* alternates between dormant spores (resistant to harsh environments) and intracellular proliferation. Upon ingestion, spores germinate and extrude a polar filament that injects the sporoplasm into host cells [53]. Subsequently, the pathogen enters its proliferative phase, and infected cells eventually lyse, releasing mature spores [54]. Our differential expression data revealed that the number of DElncRNAs increases during infection, accompanied by an overall decrease in expression levels. At 7 dpi (early invasion), TCONS_00004870 was upregulated and predicted to bind the Ricin-B lectin mRNA, which is known as a virulence factor in microsporidia [55]. At the same time, TCONS_00002929 was downregulated, correlating with the increased spore wall protein expression required for initial attachment [56,57]. At 10 dpi (proliferative phase), TCONS_00004160 positively correlated with the ATP/ADP translocase gene, the parasite’s sole machinery for stealing host ATP [49]. Both were downregulated. It is speculated that *V. ceranae* fine-tunes energy acquisition to balance replication and host cell survival. Similar metabolic reprogramming has been reported in *Nosema bombycis*-infected silkworm midguts [58]. The inoculation dose (1 × 10^6^ spores per bee) used in this study is higher than the natural infection dose typically encountered by honeybees. Although this dose was chosen to ensure consistent infection rates and sufficient parasite material for transcriptome analysis, it may have accelerated the infection cycle, potentially leading to premature lysis of infected midgut epithelial cells and release of secondary spores in 10 dpi. Consequently, the NcT2L likely contains a mixture of intracellular and extracellular secondary spores, whereas the NcT1L are predominantly intracellular. However, it should be clarified that the control group (NcCKL) represents dormant spores in vitro, whereas the infected groups (NcT1L and NcT2L) represent spores during the infection process. These DElncRNAs may be associated with infection, but could also arise from developmental differences inherent to the spores themselves (i.e., the transition from dormant spores to the mitotic stage), rather than being solely a response to host infection. In future studies, the use of in vitro-cultured spores of comparable stage, or purified mitotic-stage spores isolated under non-host conditions, may help distinguish between infection-specific lncRNA regulation and developmental expression changes.

We identified five antisense lncRNAs; one, TCONS_00004870, showed dynamic expression during infection, reminiscent of antisense-mediated gene silencing in yeast [59] and lectin-driven infectivity in *Nosema bombycis* [55]. Cis-regulation was also evident. DElncRNAs were predicted to modulate neighboring genes. Two constitutively expressed DElncRNAs (TCONS_00003691 and TCONS_00003693) shared two neighboring hypothetical protein genes, with XM_002995412.1 differentially expressed across all groups. This points to a cis-regulatory role in an essential but uncharacterized function. Moreover, TCONS_00004144—upregulated during infection—lies upstream of the *Argonaute* gene, which is an inaccessible protein in the RNAi mechanism [60]. In *trans*, multiple DElncRNAs were predicted to potentially co-target a single mRNA (e.g., six lncRNAs targeting XM_002996758.1), and one lncRNA (TCONS_00003691) can simultaneously activate one mRNA and repress another. Similarly complex trans-regulation has been seen in yeast [61] and *Trichoderma* [15]. It should be noted that these trans-regulatory predictions are derived solely from expression correlations. Functional studies are required to validate these potential interactions.

It has been proven that *V. ceranae* possesses endogenous RNA interference mechanisms and miRNA generation mechanisms [62,63,64]. The miRNAs of *V. ceranae* have been identified both in the purified spores and during the infection of the host by *V. ceranae* [65]. Meanwhile, the miRNAs of *V. ceranae* can also cross-regulate the gene expression of the host [66]. We built potential ceRNA networks where 15–23 DElncRNAs sponge 195–211 miRNAs, which in turn target 204–216 mRNAs [67]. TCONS_00003749, which targets mir-33-x—a known sporulation regulator in fungi [68]—was downregulated during infection. By reducing its sponge effect, this downregulation may enhance mir-33-x-mediated suppression of sporulation- and virulence-related genes. Target mRNAs were enriched in catalytic activity, metabolic processes, and the ubiquitin-proteasome pathway—a known regulator of cell cycle progression [69]. However, it should be noted that all these interactions are predicted and lack experimental validation. Therefore, the ceRNA network should be regarded as a hypothetical framework, and the inferred interactions may contain false positives.

Functional validation remains a major hurdle. No genetic manipulation tools exist for microsporidia [70], so our predictions rely on correlation and evolutionary conservation. The relatively small number of lncRNAs (27) might reflect both the compact genome and the stringency of our coding-potential filters. Future work should prioritize a few candidates (e.g., TCONS_00004870, TCONS_00003749) using heterologous expression or RNAi approaches when available. Despite these limitations, this study provides the first comprehensive picture of *V. ceranae* DElncRNAs and their regulatory networks (cis, trans, antisense, ceRNA). The identified lncRNAs are promising targets for functional studies and potential biomarkers for nosemosis.

## 5. Conclusions

This study presents the characterization of the lncRNA expression profile in *V. ceranae* during infection of *A. mellifera* workers. A total of 27 lncRNAs of *V. ceranae* were obtained, and the expressions of some of these lncRNAs were identified. The dynamic changes in stage-specific DElncRNAs may be related to the infection of the pathogen. Furthermore, we predicted multi-layer regulatory networks, suggesting that these DElncRNAs function through antisense, cis, trans, and ceRNA mechanisms to coordinate metabolism, cell cycle progression, and immune evasion. Overall, our findings have reported previously uncharacterized lncRNAs in microsporidia and predicted lncRNA-mediated regulatory circuits, providing novel molecular insights into *V. ceranae* pathogenesis. The key DElncRNAs identified here serve as promising targets for future functional studies using emerging genetic tools and may ultimately contribute to the development of RNA-based strategies for controlling nosemosis, thereby helping to protect honeybee health and sustain global pollination services.

## Figures and Tables

**Table 1:** Primers used in study.

Transcript ID	Primer Sequence (5′ → 3′)
TCONS_00002929	Forward: TGGATGTTGTTCCTGTGG
	Reverse: TGGACTTACGCCAATCTCTA
TCONS_00003749	Forward: TGTAGAAATACGGGCAAGC
	Reverse: TGTCACCGAAACAGGACTAA
TCONS_00003691	Forward: TGCTGTGGCTGACTGGATT
	Reverse: ATAAGAGGCAACTTCGCCG
TCONS_00002930	Forward: TGGATGTTGTTCCTGTGG
	Reverse: TGGACTTACGCCAATCTCTA
TCONS_00002069	Forward: TGTAAACGGCGGGAGTAA
	Reverse: ATGACGAGGCATTTGGCT
TCONS_00003047	Forward: CTGCTTCGCTTCATTTCC
	Reverse: GTTGGATGCCACAATACGA
TCONS_00002930	Forward: TGGATGTTGTTCCTGTGG
	Reverse: TGGACTTACGCCAATCTCTA

## Data Availability

The raw data supporting the conclusions of this article will be made available by the corresponding author on reasonable request.
